# An optimized IFN-γ ELISpot assay for the sensitive and standardized monitoring of CMV protein-reactive effector cells of cell-mediated immunity

**DOI:** 10.1186/s12865-017-0195-y

**Published:** 2017-03-07

**Authors:** Sascha Barabas, Theresa Spindler, Richard Kiener, Charlotte Tonar, Tamara Lugner, Julia Batzilla, Hanna Bendfeldt, Anne Rascle, Benedikt Asbach, Ralf Wagner, Ludwig Deml

**Affiliations:** 1 Lophius Biosciences GmbH, Am BioPark 13, 93053 Regensburg, Germany; 2 Institute of Medical Microbiology and Hygiene, University Regensburg, Franz-Josef-Strauss-Allee 11, 93053 Regensburg, Germany

**Keywords:** Cytomegalovirus, CMV, IE-1, pp65, Cell-mediated immunity, ELISpot, CD4^**+**^, CD8^**+**^, T helper (Th), Cytotoxic T lymphocyte (CTL), Natural killer (NK), NKT-like

## Abstract

**Background:**

In healthy individuals, Cytomegalovirus (CMV) infection is efficiently controlled by CMV-specific cell-mediated immunity (CMI). Functional impairment of CMI in immunocompromized individuals however can lead to uncontrolled CMV replication and severe clinical complications. Close monitoring of CMV-specific CMI is therefore clinically relevant and might allow a reliable prognosis of CMV disease as well as assist personalized therapeutic decisions.

**Methods:**

Objective of this work was the optimization and technical validation of an IFN-γ ELISpot assay for a standardized, sensitive and reliable quantification of CMV-reactive effector cells. T-activated® immunodominant CMV IE-1 and pp65 proteins were used as stimulants. All basic assay parameters and reagents were tested and optimized to establish a user-friendly protocol and maximize the signal-to-noise ratio of the ELISpot assay.

**Results:**

Optimized and standardized ELISpot revealed low intra-assay, inter-assay and inter-operator variability (coefficient of variation CV below 22%) and CV inter-site was lower than 40%. Good assay linearity was obtained between 6 × 10^4^ and 2 × 10^5^ PBMC per well upon stimulation with T-activated® IE-1 (R^2^ = 0.97) and pp65 (R^2^ = 0.99) antigens. Remarkably, stimulation of peripheral blood mononuclear cells (PBMC) with T-activated® IE-1 and pp65 proteins resulted in the activation of a broad range of CMV-reactive effector cells, including CD3^+^CD4^+^ (Th), CD3^+^CD8^+^ (CTL), CD3^−^CD56^+^ (NK) and CD3^+^CD56^+^ (NKT-like) cells. Accordingly, the optimized IFN-γ ELISpot assay revealed very high sensitivity (97%) in a cohort of 45 healthy donors, of which 32 were CMV IgG-seropositive.

**Conclusion:**

The combined use of T-activated® IE-1 and pp65 proteins for the stimulation of PBMC with the optimized IFN-γ ELISpot assay represents a highly standardized, valuable tool to monitor the functionality of CMV-specific CMI with great sensitivity and reliability.

**Electronic supplementary material:**

The online version of this article (doi:10.1186/s12865-017-0195-y) contains supplementary material, which is available to authorized users.

## Background

Human cytomegalovirus (CMV) is endemic in all human populations, with a seroprevalence ranging from 36 to 100% depending on age, gender and location [1]. In healthy individuals, CMV replication is efficiently controlled by cell-mediated immunity (CMI). In immunocompromized individuals however, the reduced frequency and functionality of CMV-reactive effector cells is associated with severe clinical complications due to uncontrolled virus replication [1–3]. Quantitative assessment of functional CMV-reactive effector cells in immunocompromized individuals might help to identify patients at increased risk for CMV-mediated clinical complications and to adjust antiviral and immunosuppressive therapy [3, 4].

Reliable monitoring of CMV-specific CMI in immunocompromized individuals, such as solid-organ or allogeneic stem cell transplant recipients, requires a specific, standardized but also highly sensitive assay capable of detecting low numbers of CMV-reactive cells. Such sensitivity might be achieved by using highly immunogenic stimulants and via the reactivation of a broad spectrum of physiological effector cells involved in the protection against CMV replication in vivo, notably CD4^+^ (T helper or Th), CD8^+^ (cytotoxic T lymphocytes or CTL), but also natural killer (NK) and natural killer T (NKT) cells [5–12].

Current diagnostic methods to detect and monitor CMV-specific CMI are mostly based on the restimulation of CD4^+^ and/or CD8^+^ effector cells with pools of overlapping peptides, cocktails of preselected immunodominant CMV-peptides or lysates of CMV-infected cells and the subsequent measurement of induced cytokine production (e.g. IFN-γ) or cell proliferation, by flow cytometry, enzyme-linked immunosorbent assay (ELISA) or enzyme-linked immunospot assay (ELISpot). A different approach consists in the direct staining with CMV-peptide-loaded multimers and enumeration of CMV-specific CD8^+^ T cells by flow cytometry. However, this method is lacking a functional readout and is restricted to certain HLA types, impeding its use in routine diagnostics.

ELISA-based assays, such as QuantiFERON®-CMV are advantageous in that they require low blood volumes and are easy to perform [13]. However, they are restricted to the detection of IFN-γ-producing CD8^+^ T cells and do not allow single-cell-level measurement. Due to analyte dilution, ELISA-based assays usually result in reduced sensitivity [14, 15] and often yield indeterminate results, especially in immunocompromized patients [16–21]. Intracellular cytokine staining (ICS) and subsequent flow cytometric analysis is usually more sensitive than ELISA [15, 20] and allows the assessment of both the functionality and the phenotype of CMV-reactivated cytokine-producing cells. However, this method is difficult to standardize and only detects the intracellular analyte, not the biologically active cytokine secreted over the stimulation period.

ELISpot-based assays identify and enumerate biologically active, cytokine-secreting cells from isolated peripheral blood mononuclear cells (PBMC), at the single-cell level both qualitatively and quantitatively [22]. ELISpot represents the most sensitive read-out system and thus is most appropriate for the detection of low-level responses [23, 24]. In particular, ELISpot is more sensitive than ICS for the detection of antigen-specific cells, such as in vitro-reactivated memory T cells, which produce only low amounts of cytokines [15, 25]. ELISpot was recently successfully employed over ELISA in the detection of congenital CMV infection [26–28].

Conventional CMV-specific ELISpot assays are based on the detection of IFN-γ-producing CD4^+^ and/or CD8^+^ T cells, depending on the antigens used for PBMC stimulation [23, 24, 29, 30]. The careful selection and formulation of antigens is crucial to ensure specificity, sensitivity and thus a diagnostic value to the assay. We have previously shown that urea-formulated recombinant (T-activated®) proteins are processed by the exogenous and endogenous antigen processing machinery, resulting in the presentation of naturally generated peptides by MHC class II and class I molecules [31]. Stimulation of PBMC by T-activated® proteins thus mimics more closely a natural infection, resulting not only in the specific activation of memory T (CD4^+^, CD8^+^) and NK cells, but also possibly in the bystander activation of NK and NKT-like cells present in the PBMC population [31, 32].

We describe here the establishment, optimization and standardization of a highly sensitive ELISpot assay that takes advantage of both the IFN-γ ELISpot readout and the immunodominant and highly immunogenic T-activated® IE-1 and pp65 CMV proteins as stimulants. We also compared the performance of the optimized assay to that of intracellular staining and flow cytometry in healthy CMV-seropositive donors, and demonstrated the ability of T-activated® IE-1 and pp65 antigens to activate a broad spectrum of CMV-reactive cells, including CD3^+^CD4^+^ (Th), CD3^+^CD8^+^ (CTL), CD3^−^CD56^+^ (NK) and CD3^+^CD56^+^ (NKT-like) cells. Therefore, the combined use of T-activated® IE-1 and pp65 CMV proteins with our optimized IFN-γ ELISpot defines a highly sensitive assay meeting all conditions for a reliable and standardized immune monitoring diagnostic tool.

## Methods

### Proteins, ELISpot plate precoating and detection conjugate

Urea-formulated T-activated® proteins were prepared as previously described [31]. The T-activated® immunodominant region of CMV pp65 (amino acids 366 to 546, hCMV strain AD169 [33]) was provided by MIKROGEN (MIKROGEN GmbH, Neuried, Germany). Full length IE-1 (hCMV towne strain) was kindly provided by Christina Paulus and Michael Nevels (University of Regensburg, Germany). T-activation® of IE-1 was performed by Lophius Biosciences. Staphylococcal enterotoxin B (SEB; 11249738001, Roche Diagnostics GmbH, Mannheim, Germany) and phytohemagglutinin (PHA; S4881, Sigma-Aldrich Chemie GmbH, Munich, Germany) were used as positive controls for PBMC stimulation. 96-well ELISpot plates (MAIPS4510, Merck Millipore, Merck KGaA, Darmstadt, Germany) and 8-well ELISpot strips (M8IPS4510, Merck Millipore) each pre-coated with anti-human-IFN-γ mAb 1-D1K (MabTech, Nacka Strand, Sweden) as well as the detection conjugate mAb-AP (7-B6-1) (MabTech, Nacka Strand, Sweden) coupled with alkaline phosphatase (Roche, Basel, Switzerland) were obtained from Microcoat Biotechnologie GmbH.

### Blood collection and PBMC preparation

Blood samples were collected in lithium heparin tubes (S-Monovette®, Sarstedt, SARSTEDT AG & Co., Nümbrecht, Germany) from healthy individuals with known CMV serostatus by venipuncture and stored for up to 8 hours at room temperature (18-25 °C) until further processing. Isolation of peripheral blood mononuclear cells (PBMC) was performed using standard Ficoll-Paque density centrifugation as specified by the manufacturer (Pancoll human, PAN-Biotech GmbH, Aidenbach, Germany). PBMC were finally suspended in serum-free AIM-V® medium (Life Technologies, Inc., Grand Island, NY) and counted either manually in a Neubauer’s chamber or using the Hem-o-test 2000 cell-counting device (BGT BioGenTechnologies GmbH, Steinfurt, Germany). Automated cell counting was performed in the whole blood venous mode of the calibrated analyzer.

### ELISpot assay

IFN-γ ELISpot assays were performed as previously described [31] unless specified otherwise in the Results section. Briefly, 2 × 10^5^ freshly isolated PBMC were plated in four replicates into 96-well ELISpot plates or 8-well ELISpot strips precoated with anti-human-IFN-γ mAb 1-D1K (Microcoat Biotechnologie GmbH) and stimulated for 19 h at 37 °C under 5% CO_2_ with either 3 μg/ml T-activated® pp65 antigen or 15 μg/ml T-activated® IE-1 protein. As unstimulated control (neg.), cells were incubated for 19 h in cell culture medium. After cell removal, plates were developed for 2 h at room temperature (18-25 °C) in the presence of 0.4 U/ml IFN-γ-specific alkaline phosphatase-coupled mAb 7-B6-1. Spot detection was performed following incubation for 6 min in the dark with a 1-step nitroblue tetrazolium–5-bromo-4-chloro-3-indolylphosphate substrate (Thermo Fischer Scientific, Waltham, USA). IFN-γ-specific spot-forming cells (SFC) were counted using a Bioreader® 5000 Eα (BIO-SYS GmbH, Karben, Germany). Of note, comparable results were obtained on two other readers (AID Elispot Robotic System ELROB05i and CTL ImmunoSpot® S6).

### Intracellular cytokine staining

Intracellular cytokine staining was performed as previously described [31], with the following modifications. PBMC from six healthy CMV-seropositive donors were isolated and four replicates each were stimulated for 6 h at 37 °C and 5% CO_2_ with 3 μg/ml T-activated® pp65 antigen or with 15 μg/ml T-activated® IE-1 protein in the presence of co-stimulatory anti-CD28 and anti-CD49d monoclonal antibodies (BD, Heidelberg, Germany). The unstimulated PBMC control cells were incubated with co-stimulatory anti-CD28 and anti-CD49d molecules only. Stimulation with SEB and anti-CD28 + anti-CD49d served as positive control. After the first two hours of incubation, 1 μg/ml brefeldin A (BFA, Sigma-Aldrich Chemie GmbH, Steinheim, Germany) was added to prevent cytokine secretion from activated cells. Surface markers (CD3, CD4, CD8, CD56) were stained for 30 min at 4 °C using the following conjugated antibodies (all from Biolegend, London, UK): anti-CD3 APC-Cy7, anti-CD4 Brilliant Violet 421, anti-CD8 FITC, anti-CD56 PerCP. Cells were fixed and permeabilized for 30 min at 4 °C in BD Cytofix/Cytoperm (BD, Heidelberg, Germany). Intracellular staining of IFN-γ was performed using anti-IFN-γ APC (Biolegend, London, UK) in BD Perm/Wash Buffer (BD, Heidelberg, Germany) for 45 min at 4 °C. Samples were analyzed on a BD FACSCanto™ II flow cytometer (Becton Dickinson, USA). Live-gating of cells was performed during acquisition. Mean (SD) number of event acquisition was 172,904 (108,298). Results were reported as percentage of the gated population producing IFN-γ (dot plots) and as the number of IFN-γ^+^ cells/200,000 lymphocytes (bar graphs).

### Serology

The CMV serological testing of blood donors was performed using the fully automated CMV immunoglobulin M (IgM) and IgG tests on the Architect instrument (Abbott Laboratories, Abbott Park, IL) or the BEP® III system (Siemens Healthcare) by the diagnostics department of the Institute for Medical Microbiology and Hygiene (University of Regensburg, Germany). CMV IgG-serology was used as primary reference measurement procedure (gold standard method).

### Statistics

**Fig. 1:**
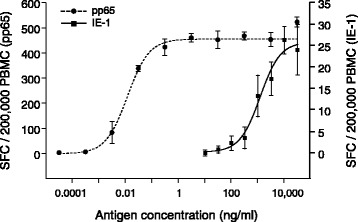
Determination of optimal concentrations of T-activated® pp65 and IE-1 for the stimulation of PBMC. PBMC were isolated from one CMV-seropositive healthy donor blood sample and stimulated for 19 h with increasing concentrations of T-activated® pp65 (31.6 fg/ml-31.6 μg/ml; black circles) or IE-1 (0.01-31.6 μg/ml; black squares). IFN-γ ELISpot results of four replicates are expressed as mean SFC/200,000 PBMC. Error bars represent standard deviations. The Y-axis scale was adjusted to pp65- (left) and IE-1- (right) specific values to optimize data resolution. Curve fitting was made using the four parameter logistic function of GraphPad Prism 5.04

Statistics applied to assay development was performed using SigmaPlot Version 11.0 and GraphPad Prism 5.04. At the start of this study, a positive ELISpot test result was defined by a statistically significant difference between quadruplicate SFC values of non-stimulated and specifically stimulated approaches of at least one of the two antigens used, calculated using the Mann–Whitney *U*-Test (MWU) (SigmaPlot). Two-sided exact *P* values are reported. *P* values < 0.05 were considered statistically significant. Statistical analyses to assess variations between two or more settings were performed by generating arithmetic means for all replicates of each given setting. Those means were grouped to generate an overall arithmetic mean and standard deviation. The coefficient of variation in % (ratio of the standard deviation to the mean multiplied by 100) was calculated in Microsoft Excel. The curve fitting for sigmoidal curves of the titration experiment (Fig. 1) was performed using the four parameter logistic function of GraphPad Prism 5.04. For the assessment of the ELISpot assay sensitivity in seropositive healthy blood donor collectives, the non-parametric Mann–Whitney *U*-Test (MWU test) was performed to determine if a significant difference exists between the groups of negative control and stimulation quadruplicates. Multiple-group comparisons were performed using a non-parametric One-way ANOVA (Kruskal-Wallis test) with Dunn’s multiple comparison post test. SFC values were depicted using GraphPad Prism 5.04 either as Tukey box plots showing median values (horizontal line), interquartile ranges (IQR: Q3-Q1), lower and upper whiskers (Q1-1.5xIQR and Q3 + 1.5xIQR respectively) and outliers (below Q1-1.5xIQR and above Q3 + 1.5xIQR; black dots), or as histograms. For assay validation of optimized ELISpot, positivity cut-off was calculated on IFN-γ ELISpot results obtained from a collective of 45 healthy donors using SAS 9.2 Software and VFP (Variance Function Program; option “Simple variance function estimate”) version 12.0.

## Results

### *IFN-γ ELISpot assay optimization following PBMC stimulation with* T-activated® IE-1 and pp65 CMV antigens

Freshly isolated PBMC were used for the ELISpot assay. To prevent loss in T cell functionality, for instance due to activated granulocytes [34], heparinized blood samples were processed with no further additives within 8 h. A total number of 2 × 10^5^ PBMC per well was chosen for the development of the ELISpot assay protocol as this cell count is below confluency and can usually be obtained from samples of less than 15 ml whole blood. The CMV immediate-early protein IE-1 and the late tegument protein pp65 represent well-characterized immunodominant T cell antigens [1, 24, 35]. Full-length IE-1 and a 181 amino-acid C-terminal fragment of pp65 were produced and formulated in the presence of urea (T-activation®) to increase their stimulatory capacity for different types of CMV-reactive effector cells of cell-mediated immunity [31]. Optimal T-activated® antigen concentration was first determined by performing dose–response experiments. Freshly isolated PBMC of one healthy CMV-seropositive donor were stimulated with 31.6 fg/ml to 31.6 μg/ml T-activated® pp65 or with 0.01 to 31.6 μg/ml T-activated® IE-1, and the number of IFN-γ secreting cells was determined by IFN-γ ELISpot. T-activated® pp65 revealed a much stronger capacity to stimulate IFN-γ secreting effector cells than T-activated® IE-1, reaching a plateau of responsiveness between 0.316 and 3.16 ng/ml pp65 vs. approximately 31.6 μg/ml for IE-1 (Fig. 1). Accordingly, T-activated® antigen concentrations of 3 μg/ml pp65 and 15 μg/ml IE-1 were selected for further PBMC stimulations and ELISpot assays.

**Fig. 2:**
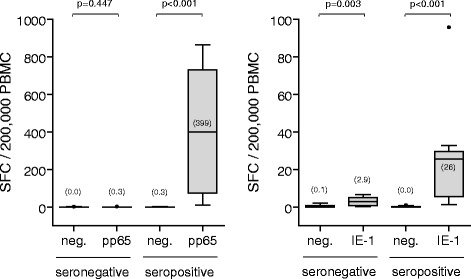
Determination of assay sensitivity and specificity. PBMC from 10 each CMV-seropositive and CMV-negative healthy blood donors were left unstimulated (neg.) or were stimulated with T-activated® pp65 or IE-1 and IFN-γ ELISpot assays were conducted as before. Mean SFC values per 200,000 PBMC of 4 replicates are shown as box plots. Median values (horizontal black lines) are indicated in brackets. Y-axis scales were adjusted in each graph for better resolution of SFC counts. Median age and range of CMV-seronegative and CMV-seropositive subjects was 28 (24–53) and 31 (23–56) years. Gender distribution in CMV-seronegative (30% male and 70% female) and CMV-seropositive (25% male and 75% female) groups was comparable. Differences between unstimulated and stimulated conditions were tested using the non-parametric two-sided Mann–Whitney U (MWU) test. P-values < 0.05 were considered statistically significant

Assay sensitivity and specificity were determined by stimulating PBMC isolated from 10 each CMV-seropositive and CMV-seronegative healthy donors with the defined pp65 and IE-1 T-activated® antigen concentrations. The number of reactive effector cells was quantified by IFN-γ ELISpot. Significant stimulation was defined using a Mann–Whitney *U*-Test as a statistically significant difference between SFC values of non-stimulated and CMV antigen-stimulated conditions (each in quadruplicate). T-activated® pp65 and IE-1 induced a significant activation of responsive effector cells in 10 out of 10 and 9 out of 10 PBMC preparations from individual CMV-seropositive donors, respectively (Fig. 2). In this collective, T-activated® pp65 showed an overall greater capacity to activate responsive cells with a median of 399 SFC/200,000 PBMC (range 12–864 SFC/200,000 PBMC), compared to T-activated® IE-1 with a median of 26 SFC/200,000 PBMC (range 1.3-96 SFC/200,000 PBMC). Nevertheless, substantial response of up to 96 SFC/200,000 PBMC was detected in response to T-activated® IE-1 in individual samples of CMV-seropositive donors (Fig. 2). All 10 PBMC samples (100%) from different CMV-seronegative donors showed negative test results after stimulation with pp65 (median 0.3 SFC/200,000 PBMC; range 0–2.8), while 9 out of 10 (90%) PBMC samples from CMV-seronegative individuals were negative after stimulation with IE-1 (median 2.9 SFC/200,000 PBMC; range 0.3-6.8). Spot count within CMV-seronegative IE-1-stimulated PBMC was higher than in CMV-seronegative pp65-stimulated PBMC but did not exceed 7 SFC/200,000 PBMC (Fig. 2).

**Fig. 3:**
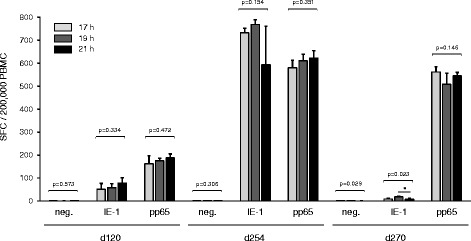
Effect of duration of antigen stimulation on IFN-γ ELISpot test results. SFC counts (means of four replicates) in IFN-γ ELISpot following stimulation of PBMC samples from three CMV-seropositive healthy donors (d120, 32-year-old male; d254, 62-year-old female; d270, 22-year-old female) with T-activated® IE-1 or pp65 for 17, 19 and 21 h. Unstimulated PBMC (neg.) were used as a negative control. Differences between stimulation durations were tested using the non-parametric two-sided One-way ANOVA Kruskal-Wallis test (*P < 0.05). P-values < 0.05 were considered statistically significant

IFN-γ has been shown to be secreted continuously during antigen stimulation. Thus, signal intensity in IFN-γ ELISpot is dependent on the duration of stimulation [14, 36]. The duration of antigen stimulation in IFN-γ ELISpot reported in the literature usually ranges from 16 to 24 h [37–42]. To address the influence of the incubation time on the test results, IFN-γ ELISpot were performed on PBMC from 3 independent CMV-seropositive healthy donors following stimulation with T-activated® pp65 and IE-1 antigens for 17, 19 and 21 h. No statistically significant differences in SFC numbers were detected between the 3 conditions (Fig. 3), demonstrating signal stability in this time range. Thus, an incubation time of 19 h was chosen for the optimized ELISpot assay.

**Fig. 4:**
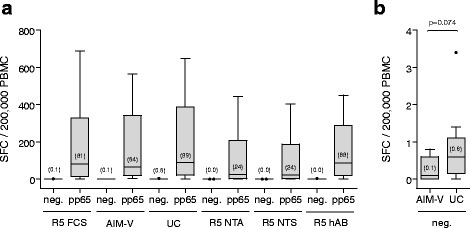
Evaluation of different cell culture media on the performance of IFN-γ ELISpot. **a** Boxplot diagram showing ELISpot results upon stimulation of PBMC of 10 CMV-seropositive healthy blood donors (median age and range of 31 (26–54) years; 40% male and 60% female) with T-activated® pp65 in various cell culture media. Median values (horizontal black lines) are indicated in brackets. Differences between medium conditions among stimulated conditions were tested using the non-parametric two-sided One-way ANOVA Kruskal-Wallis test (p = 0.613). **b** ELISpot results of the non-stimulated cells incubated in AIM-V or in UltraCulture (UC) serum-free media are shown separately with an expanded Y-axis scale. Differences between both conditions were tested using the non-parametric two-sided MWU test (p = 0.074). P-values < 0.05 were considered statistically significant. Serum-containing media were composed of RPMI 1640 supplemented with 5% serum (R5): FCS, NTA, NTS or human AB (hAB)

Numerous protocol variables can affect ELISpot test results. For instance, the medium used for primary cell culture often includes serum which contains various batch-dependent non-characterized bioactive molecules in different concentrations [43]. In order to define standardized cell culture conditions, the impact on the assay performance of different serum-containing media (RPMI 1640 supplemented with 5% of either FCS, human AB, synthetic NTA or synthetic NTS) and of serum-free media (AIM-V®, UltraCulture) was investigated. Serum-free media yielded the best effector cell responses, comparable to that of RPMI 1640 supplemented with 5% FCS (Fig. 4a). In addition, AIM-V® exhibited lowest background signals in unstimulated conditions (Fig. 4b), thus maximizing signal-to-noise ratio. Consequently, the ELISpot protocol was further established using AIM-V® serum-free medium.

**Fig. 5:**
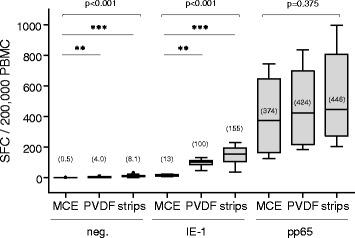
Comparison of IFN-γ ELISpot performance using different microtiter plates. ELISpot were performed using PBMC from three CMV-seropositive healthy donors (two preparations each, ELISpot in quadruplicate, ran twice) on various microtiter plate materials (MCE plate, PVDF plate, PVDF strips). Age (gender) of donors was 32 (male), 33 (male) and 51 (female). SFC values per 200,000 PBMC are shown as box plots for non-stimulated PBMC (neg.), and for T-activated® IE-1- and pp65-stimulated PBMC. Median values (horizontal black lines) are indicated in brackets. Differences between microtiter plate materials were tested using the non-parametric two-sided One-way ANOVA Kruskal-Wallis test (**P < 0.01; ***P < 0.001)

ELISpot assays can be performed with various membrane materials, including nitrocellulose (NC), mixed cellulose ester (MCE) and polyvinylidene fluoride (PVDF). We compared IFN-γ ELISpot results from PBMC samples of six healthy individuals (four replicates each, two preparations per donor) employing the most commonly used MCE plates, PVDF plates and PVDF strips from Millipore (Merck Millipore, Merck KGaA, Darmstadt, Germany). PVDF membranes require an activation step with ethanol prior to binding of the capture antibody. In addition, more stringent washing steps prior to spot development were needed using PVDF-based plates compared to MCE plates, to avoid undesirable background staining of membranes. Nevertheless, the resolution of the detected spots in terms of sharpness and homogeneity was improved on PVDF membranes (not shown), resulting in higher spot counts compared to MCE membranes (up to 10-times more in the case of IE-1 stimulations, with a median SFC of 155 vs. 13/200,000 PBMC, respectively; Fig. 5). Since PVDF 8-well strips were more performant and because their use might allow reduced costs, in particular when single patient samples are tested in clinical routine, the PVDF 8-well-strip format was chosen for the optimized assay development.

**Fig. 6:**
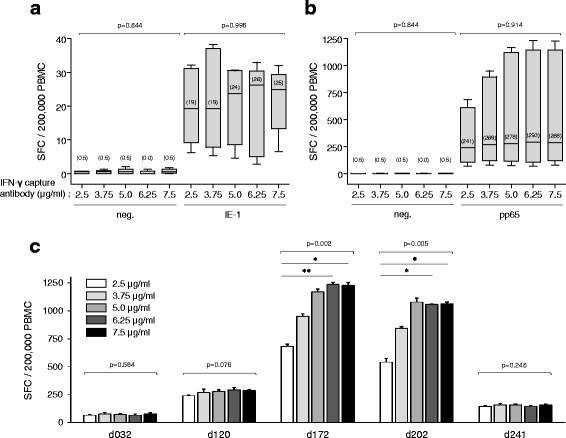
titration of the ifn-γ capture antibody. PBMC (four replicates each) from five CMV-seropositive healthy donors (median age and range of 31 (22–49) years; 1 male and 4 female) were seeded on PVDF microtiter strips coated with increasing concentrations (2.5 to 7.5 μg/ml) of IFN-γ capture antibody. Cells were left unstimulated (neg.) (**a, b**) or were stimulated with T-activated® IE-1 (**a**) or pp65 (**b, c**) CMV antigens, as before. SFC mean values per 200,000 PBMC are shown as box plots for the collective of 5 donors (median values indicated in brackets) (**a, b**) and as histograms (**c**) for pp65-stimulated PBMC of individuals donors (d032, d120, d172, d202, d241). Differences between coating conditions were tested using the non-parametric two-sided One-way ANOVA Kruskal-Wallis test (*P < 0.05; **P < 0.01)

Optimal coating of microtiter plates with capture antibody is crucial for a robust assay performance. The density of IFN-γ capture antibody bound to the PVDF membrane should not be limiting and, in particular, should allow the reliable detection of high spot counts. PVDF strips were coated with increasing concentrations (2.5 to 7.5 μg/ml) of anti-IFN-γ antibody. PBMC from five CMV-seropositive donors were seeded into the coated wells and either left unstimulated or stimulated with T-activated® IE-1 or pp65. In the range of 2.5 to 7.5 μg/ml, increasing antibody concentrations had no effect on background staining in unstimulated PBMC (median SFC of 0 to 0.5/200,000 PBMC regardless of IFN-γ capture antibody concentration; Figs. 6a-b). Similarly, IE-1-specific low-to-moderate SFC levels (medians of 19 to 26 SFC/200,000 PBMC) were comparable in all coating-antibody conditions (Fig. 6a). The same was true for pp65-specific responses up to ~300 SFC/200,000 PBMC (donors d032, d120 and d241; Fig. 6c). In contrast, in individual donors with higher spot counts (e.g., d172, d202; Fig. 6c), detection of pp65-reactive cells increased in a dose-dependent manner with the concentration of IFN-γ capture antibody used for coating, especially between 2.5 and 5 μg/ml antibody. At antibody concentrations of 5 μg/ml and beyond, spot counts remained stable (Figs. 6b-c). Based on these results, a concentration of 5 μg/ml IFN-γ capture antibody was chosen for coating in the optimized ELISpot protocol.

ELISpot assays rely on the detection of captured cytokine by cytokine-specific antibodies, which can be either directly coupled to a reporter enzyme (one-step assay development), like alkaline phosphatase (AP), or a combination of a biotinylated secondary antibody and a streptavidin-conjugated reporter enzyme (two-step assay development). A one-step assay development saves handling time and prevents operator errors. AP-conjugated mAb (7-B6-1) (MicroCoat Biotechnologie GmbH, Bernried, Germany) was used as detection conjugate for the standardized ELISpot protocol. Different incubation parameters (e.g., 0.5 – 3 h at 37 °C, 2 h at room temperature [RT, 18-25 °C]) were tested. Spot counts were comparable in all conditions. However, spot morphology and thus proper detection was best upon incubation with AP-conjugate for 2 h at RT, compared to other conditions (not shown). Increase in the concentration of the detection conjugate from 0.025 to 0.4 U/ml resulted in slightly elevated SFC median values for pp65, and the maximum spot counts exceeded those generated by the two-step assay development (not shown). Therefore, a one-step assay development for 2 h at RT with 0.4 U/ml detection conjugate was chosen.

Finally, standardization of the SFC staining reaction was addressed to complete assay optimization. Duration of incubation with the AP substrate affects spot size and/or background level, and is thus critical for reliable spot enumeration. A one-step chromogenic alkaline phosphatase substrate NBT/BCIP (Thermo Fischer Scientific, Waltham, USA) was used as staining solution, and incubation times ranging from 2 to 13 min in the dark were evaluated. SFC counts were comparable in all conditions. However, shorter incubation times resulted in smaller spot diameter, while longer incubation times yielded enhanced background levels (data not shown). Therefore, a staining duration of six minutes in the dark was defined for the optimized ELISpot protocol.

### Linearity and precision of the optimized CMV ELISpot assay

**Fig. 7:**
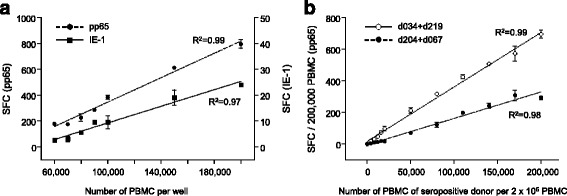
IFN-γ ELISpot assay linearity. **a** Working range of PBMC per ELISpot assay**.** Increasing number of PBMC from one CMV-seropositive healthy donor were seeded per well and ELISpot assays were performed as described, following stimulation with T-activated® IE-1 or pp65. Mean SFC values and standard deviation obtained for 60,000-200,000 PBMC per well are depicted. Scale of the Y-axes was adjusted for pp65 (left) and IE-1 (right) for a better data resolution. **b** Linearity between the number of CMV-reactive PBMC and enumerated SFC. The indicated numbers of PBMC from one CMV-seropositive healthy donor were mixed with PBMC from one CMV-seronegative donor (up to 200,000 total PBMC) and stimulated with T-activated® pp65 antigen according to the optimized protocol. Mean SFC values and standard deviation of quadruplicate measurements are shown for two donor pairs (d034 + d219, d204 + d067). In both panels, regression lines and corresponding coefficient of determination R^2^ were generated using the regression line tool of GraphPad Prism 5.04

The optimized ELISpot protocol was used to determine the working range of PBMC that ensure assay linearity. PBMC from one healthy CMV-seropositive donor were seeded at a density ranging from 2 × 10^4^ to 2.5 × 10^5^ PBMC per well. For cell numbers between 6 × 10^4^ and 2 × 10^5^ PBMC per well, ELISpot counts were directly proportional to the number of PBMC seeded, following stimulation with either IE-1 (linear regression analysis; R^2^ = 0.97) or pp65 (R^2^ = 0.99) (Fig. 7a). Because of the usually lower spot count resulting from IE-1 stimulation, the use of 2 × 10^5^ PBMC per well was chosen for the standardized ELISpot assay, to ensure sufficient SFC values.

To further verify the linearity between the number of CMV-reactive effector cells and enumerated SFC, increasing numbers of PBMC from one CMV-seropositive healthy donor were seeded and the total number of PBMC was adjusted to 2 x 10^5^ per well using PBMC from one CMV-seronegative donor. Two donor pairs (d034 + d219, d204 + d067) that showed no allo-reactivity in a 19-h co-culture were chosen for these experiments. Due to low SFC numbers (below 20 SFC/200,000 PBMC), IE-1 stimulation results showed increased variability compared to pp65 and did not allow a reliable linearity calculation (R^2^ values below 0.96; data not shown). ELISpot assay results following pp65 stimulation showed a good linear correlation for both donor pairs, with R^2^ values of 0.99 (d034 + d219) and 0.98 (d204 + d067) in the linear regression analysis (Fig. 7b).

Precision and repeatability of the optimized assay were evaluated by calculating the intra-assay, inter-assay, inter-operator and inter-site variability. In each case, PBMC from three CMV-seropositive healthy donors were tested in quadruplicate, and variability was assessed by calculating the coefficient of variation (CV), defined as the ratio of the standard deviation to the mean. CV for ELISpot values < 10 SFC/200,000 PBMC were not calculated (see positivity cut-off calculation below). CV intra-assay reached 14% for IE-1 stimulation and 6% for pp65 stimulation (Additional File 1: Table S1). CV inter-assay did not exceed 17% for IE-1 and 22% for pp65 (Additional File 1: Table S2). CV inter-operator was below 13% and 18% for IE-1 and pp65 respectively (Additional File 1: Table S3). Finally, inter-site variation, which is essential for assay validation for diagnostics purposes, was evaluated. Whole blood samples were simultaneously collected from three CMV-seropositive healthy donors and shipped (under constant condition at RT) to four different laboratories in Germany. PBMC were freshly isolated and ELISpot assays performed according to the optimized protocol by a total of 7 operators. CV inter-site reached a maximum of 39% for IE-1 and of 28% for pp65 (Additional File 1: Table S4).

Evaluation of at least four independent measurements has been recommended to achieve statistically significant ELISpot results [44–46]. To address the influence of intra-replicate variations on the assay outcome, we compared ELISpot results of quintuplicate and quadruplicate measurements of each control and antigen stimulations. No significant variation of test results was found (data not shown). Therefore, quadruplicate measurements of unstimulated and T-activated® IE-1- and pp65-stimulated conditions allow assay reliability, as well as practicability in combination with the use of 8-well strips.

Staphylococcal enterotoxin B (SEB) is a powerful superantigen, and phytohemagglutinin (PHA) a potent mitogen, both inducing massive IFN-γ secretion by T cells [47, 48]. Thus SEB and PHA are suitable positive controls for cell viability, successful stimulation of cytokine secretion and overall T cell functionality. This is particularly important for result interpretation when low T cell frequency is expected, for instance in recipients of allogeneic stem cell transplantation. In the optimized ELISpot assay, stimulations of test samples with either SEB or PHA are performed in duplicate.

In addition, an effector cell-independent operator control was established to validate proper assay performance. This operator control is based on the detection of recombinant IFN-γ upon direct incubation with the immobilized anti-human-IFN-γ mAb (1-D1K) capture antibody. This control, also performed in duplicate, should yield a homogeneous dark staining of the PVDF membrane.

### Technical assay validation: definition of a positivity cut-off

To ease result interpretation of the IFN-γ ELISpot assay and to maximize specificity (i.e. avoid false positives within unstimulated conditions and within stimulated conditions in CMV-seronegative individuals), a technical cut-off was defined. IFN-γ ELISpot assays were performed according to the optimized protocol on PBMC from 45 healthy donors, of which 32 were CMV IgG-seropositive (Table 1).

**Table 1:** Technical validation cohort of healthy donors

Median age (range) in years	33 (21; 64)
Gender, N (%)	
Male	11 (24.4%)
Female	34 (75.6%)
CMV serostatus, N (%)	
Positive	32 (71.1%)
Negative	13 (28.9%)

**Fig. 8:**
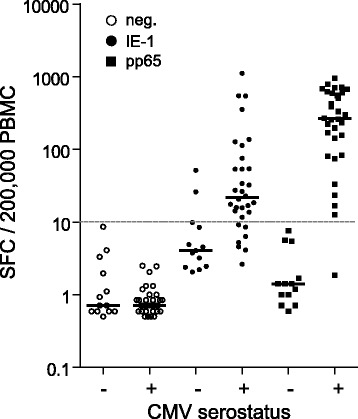
Assay validation in immunocompetent donors. PBMC isolated from whole blood of 45 healthy donors (Table 1) were assayed using the optimized IFN-γ ELISpot. A positivity cut-off of 10 SFC/200,000 PBMC (grey horizontal dashed line) was defined (see text and Additional File 2). Considering a test result as positive when the geometric mean for at least one of the IE-1 or pp65 stimulated approach is ≥ 10 SFC/200,000 PBMC and when the ratio of geometric means of stimulated to unstimulated conditions is ≥ 2.5, positive agreement (sensitivity) and negative agreement (specificity) of the optimized IFN-γ ELISpot test results with CMV serology within this collective of healthy donors was 97% and 85% respectively

Median and range of SFC values from unstimulated PBMC of CMV-seronegative and CMV-seropositive individuals were comparable [median SFC (range) of 0.7 (0.5-8.6) and 0.7 (0.5-2.5)/200,000 PBMC, respectively; Fig. 8]. Spot counts in IE-1- and pp65-stimulated PBMC of CMV-seronegative subjects were low [median SFC (range) of 4.0 (2.0-52) and 1.4 (0.6-7.6)/200,000 PBMC, respectively; Fig. 8]. In CMV-seropositive individuals, SFC levels in IE-1- and pp65-stimulated PBMC reached 1114 and 954 spot counts/200,000 PBMC (median of 22 and 265 respectively; Fig. 8). For the determination of a technical cut-off, spot counts in the unstimulated control as well as in T-activated® pp65- and IE-1-stimulated conditions of CMV-seronegative and CMV-seropositive individuals were taken into consideration. Positivity threshold was determined using z-statistics (α-level = 0.05) on log10-transformed geometric mean values. Values = 0 were replaced by values near detection limit, which was assumed to be 0.5. Standard deviation (SD) of ELISpot measurements for the unstimulated control, IE-1 stimulation and pp65 stimulation was respectively 0.234, 0.192 and 0.136. Considering a SD of 0.2 and assuming that 4 replicates are measured for each negative control and test samples, a criterion that the ratio of geometric means of stimulated to unstimulated values is at least 2.5 was calculated. On the other hand, precision profiles were generated from both IE-1- and pp65-specific test results, whereby a coefficient of variation (CV) no higher than 40% was used as a limit of acceptance of assay validity to determine the respective limit of quantitation (LoQ). Precision profiles for IE-1- and pp65-specific ELISpot results from the 45 healthy donors yielded LoQ values of 8.6 and 7.1 respectively (Additional File 2). Of note, a similar analysis performed on a cohort of 124 hemodialysis patients provided comparable SD values within unstimulated and antigen-stimulated conditions (range 0.199-0.240) and yielded LoQ values of 7.8 (IE-1) and 8.3 (pp65) [49]. Based on these analyses, a cut-off of 10 SFC/200,000 PBMC was chosen to define positive test results.

Altogether, using T-activated® pp65 and IE-1 antigens and the optimized IFN-γ ELISpot assay, test results are considered positive if the geometric mean of the spots resulting from pp65 or IE1 stimulations are ≥ 10 SFC/200,000 PBMC and if the ratio of the geometric means of stimulated to non-stimulated conditions is ≥ 2.5. According to these definitions, the collective of 45 healthy donors (32 CMV IgG-seropositive, 13 CMV IgG-seronegative) revealed a sensitivity (defined as the positive agreement with CMV IgG-serology, used as primary reference measurement procedure) of 97% and a specificity (negative agreement with CMV IgG-serology) of 85% (Fig. 8).

### Functional assay validation: T-activated antigens stimulate a broad spectrum of clinically relevant CMV-reactive effector cells

**Fig. 9:**
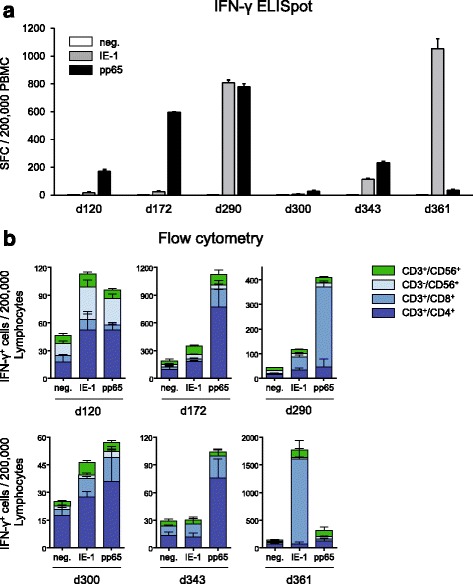
T-activated® pp65 and IE-1 CMV antigens stimulate a broad range of CMV-reactive effector cells. Comparative analysis of IFN-γ secreting cells by ELISpot (**a**) and flow cytometry (**b**) following stimulation of PBMC with T-activated® pp65 and IE-1 antigens. PBMC (4 replicates each) from six CMV-seropositive healthy donors (median age and range of 39 (22–55) years; 2 male and 4 female) were stimulated with T-activated® pp65 and IE-1 antigens according to the optimized IFN-γ ELISpot (**a**) and to the protocol of intracellular and surface marker staining and flow cytometry described in the Methods section (**b**). Bar graphs in (**a**) depict IFN-γ-dependent SFC per 200,000 PBMC, as before. Bar graphs in (**b**) represent the number of IFN-γ-expressing CD3^+^CD4^+^ (Th), CD3^+^CD8^+^ (CTL), CD3^−^CD56^+^ (NK) and CD3^+^CD56^+^ (NKT-like) cells per 200,000 lymphocytes. Note that the Y-axis scales in (**b**) were adjusted for a better resolution of the respective data. Individual age and gender of donors were as follows: d120, 37-year-old male; d172, 55-year-old female; d290, 46-year-old female; d300, 41-year-old female; d343, 24-year-old female; d361, 22-year-old male

Urea-formulated recombinant (T-activated®) proteins are processed by both the exogenous (MHC class II) and endogenous (MHC class I) antigen processing and presentation pathways [31]. Stimulation of PBMC by T-activated® proteins thus reproduces more closely a natural infection, potentially resulting in the activation of a broad spectrum of clinically relevant CMV-reactive cells (e.g., Th, CTL, NK, NKT cells; [5–12]), which might contribute to the high sensitivity of the IFN-γ ELISpot assay. To further characterize the cells targeted by T-activated® IE-1 and pp65 antigens and to investigate the possible inter-individual variability in the effector cells response, intracellular IFN-γ staining and flow cytometry analyses were performed in parallel to IFN-γ ELISpot assays. Freshly isolated PBMC from six CMV-seropositive healthy donors were stimulated with T-activated® IE-1 and pp65 proteins for 19 h and IFN-γ-producing cells enumerated according to the optimized ELISpot assay. The same PBMC preparations (4 replicates each) were stimulated for 6 h with the same batch of T-activated® IE-1 and pp65 proteins in the presence of co-stimulatory anti-CD28 and anti-CD49d antibodies. Surface markers (CD3, CD4, CD8, CD56) and intracellular IFN-γ staining were analysed by flow cytometry, as described in the Methods section. IFN-γ^+^ subpopulations of CD3^+^CD4^+^ (Th), CD3^+^CD8^+^ (CTL), CD3^+^CD56^+^ (NKT-like) and CD3^−^CD56^+^ (NK) lymphocytes were enumerated following the gating strategy illustrated in Additional File 3. All six donors differed in their capacity to elicit an IE-1- and/or pp65-dependent response in the IFN-γ ELISpot assay. The intensity of the response was also heterogeneous among the six donors, with values ranging from 7 to 1,054 SFC (IE-1 stimulation) and from 28 to 780 SFC (pp65 stimulation) per 200,000 PBMC (Fig. 9a). Similarly, individual donors differed in the frequency and ratio of the various cell subpopulations investigated by flow cytometry (Fig. 9b). Interestingly, healthy CMV-seropositive individuals with lower spot counts (d120, d300, d343; Fig. 9a) also showed lower frequencies of IFN-γ^+^ lymphocytes by flow cytometry (Fig. 9b), highlighting the ability of the ELISpot assay to distinguish low from high responders. Activation of each lymphocyte subpopulation investigated was detected in some but not all donors. For instance, weak to strong CD4^+^ T cell activation was detected in 4 out of 6 donors (d120, d172, d300, d343) in response to either IE-1, pp65, or both. Similarly, 5 out of 6 donors (d172, d290, d300, d343, d361) showed CD8^+^ T cell activation in one or both stimulation conditions. CD3-CD56^+^ (NK cells) were apparent in one donor (d120) in response to both antigens. CD3^+^CD56^+^ (NKT-like) cells were weakly activated in 4 out of 6 individuals (d120, d172 d300, d361) in response to both IE-1 and pp65 (Fig. 9b). Remarkably, a strong pp65-specific CD4^+^ activation in d172 correlated with a strong pp65-specific response in ELISpot, and a strong pp65-specific (d290) and IE-1-specific (d361) CD8^+^ activation was associated with a high spot count in the corresponding ELISpot (Figs. 9a-b), suggesting that these CMV-reactive effector cells significantly contribute to the detected ELISpot signals. Altogether, these data demonstrate the ability of T-activated® IE-1 and pp65 proteins to stimulate a broad range of CMV-specific effector cells. These experiments also revealed the high heterogeneity of responses among healthy CMV-seropositive individuals. Notably, the observation that some individuals respond to either IE-1 or pp65 emphasizes the importance of assessing the response to both antigens. Monitoring both IE-1- and pp65-specific responses in the optimized IFN-γ ELISpot assay might improve the overall sensitivity of the test.

## Discussion

We describe here the development and technical validation of an optimized and standardized IFN-γ ELISpot protocol for the monitoring of CMV-reactive effector cells of cell-mediated immunity (CMI).

The particularity of the assay is the use of T-activated® proteins [31] as stimulant of CMV-reactive effector cells. We now demonstrate that urea-formulated IE-1 and pp65 proteins are capable of activating not only CMV-reactive CD4^+^ (Th) and CD8^+^ (CTL) lymphocytes, but also innate lymphocytes (CD3^−^CD56^+^ NK and CD3^+^CD56^+^ NKT-like cells), likely by bystander activation as well as by activation of CMV-specific memory NK cells [5, 7, 8, 32, 50, 51]. This finding is important given the acknowledged role played by these effector cells in the protection against CMV reactivation in vivo [5–12]. Inter-individual variability in the response of lymphocyte subpopulations was high among CMV-seropositive healthy donors, therefore further emphasizing the importance of an antigen formulation capable of activating a broad spectrum of CMV-reactive effector cells. In this, the optimized IFN-γ ELISpot assays outperforms existing ELISpot- and ELISA-based CMV-specific CMI monitoring tools. In addition, the characteristics and high sensitivity of our assay predict a performance independent of the HLA-type of the donor.

The second particularity of the described assay is the combined use of two T-activated® proteins, IE-1 and pp65, as antigens. We show here that both antigens can elicit the activation of distinct CMV-specific effector cells and that while some CMV-seropositive healthy individuals are reactive to both antigens, some are only reactive to one (either IE-1 or pp65), as previously reported [52]. Therefore, consideration of both test results is expected to improve the sensitivity of the assay, notably in cohort studies. This is particularly relevant for the monitoring of CMV-specific CMI in immunocompromized patients. Accordingly, we recently demonstrated the benefit of measuring the response to both T-activated® IE-1 and pp65 antigens in terms of assay sensitivity, in clinical studies assessing CMV-specific CMI during pregnancy [53], in hemodialysis patients [49] and in renal transplant recipients (submitted).

The assessment of the response to both IE-1 and pp65 antigens is further justified by the respective relevance and differential contribution of IE-1- and pp65-reactive effector cells to the protection against CMV reactivation and related clinical complications, both in healthy CMV-seropositive individuals and in transplant recipients [1, 6, 54–59]. The differential contribution of IE-1- and pp65-specific effector cells likely reflect the dynamic of the cell-mediated immune response [10, 60–64]. Mechanisms of immune evasion inhibiting MHC-I-dependent IE-1 antigen processing and presentation might also explain the lower frequency of IE-1-specific IFN-γ-producing cells detected in our ELISpot assay [65]. In addition, reduced processing and presentation efficiency due to protein stability, size and nuclear localization might play a role in the reduced reactivity to IE-1, as opposed to pp65 [64, 66, 67]. This proposition is supported by the observation that higher concentrations of IE-1 were required in our ELISpot assay, in comparison to pp65, to trigger a significant response.

Another particularity of our IFN-γ ELISpot assay was its ability to elicit positive test results in CMV-seronegative individuals, notably following stimulation with IE-1. In the cohort of 45 healthy donors, out of the 13 CMV-seronegative individuals 2 showed IE-1-specific values ≥ 10 SFC/200,000 PBMC (Additional File 2), corresponding to a negative agreement with CMV IgG-serology of 85%. Similar results were obtained in a cohort of 124 hemodialysis patients [49]. Numerous studies reported discordant results between cellular and humoral immunity against CMV [68–71]. Given the high sensitivity of our ELISpot assay and its ability to detect a broad range of CMV-reactive cells, it is possible that cellular reactivity in CMV-seronegative individuals reflects a previous exposure to CMV that failed to mount a humoral immune response, rather than a false-positive ELISpot result. Further experiments will be necessary to address this possibility. Nonetheless, this observation raises the question of the accuracy of CMV serology to identify immunocompromised patients at increased risk of CMV reactivation [3, 70, 72].

The optimized and standardized IFN-γ ELISpot assay exhibited robust performance in terms of assay variability, precision and linearity. Intra-assay, inter-assay and inter-operator coefficients of variations (CV) did not exceed 22%, which is below the range of recommended precision (% of relative standard deviation or %RSD) for cell-based assays [73]. As predicted, inter-site variability involving different operators was higher, but remained acceptable with a CV of no more than 39%. This is an important criterion for the reliable longitudinal monitoring of CMV-specific immune responses in patients. Linearity of the assay is also a critical parameter, in particular for the monitoring of immunocompromized patients with reduced and/or functionally impaired T cells, such as children or recipients of allogeneic stem cell transplantation. Good linearity and high signal-to-noise ratio were obtained for both IE-1 (R^2^ = 0.97) and pp65 (R^2^ = 0.99) in the range of 60,000 to 200,000 PBMC per well. Two multi-center clinical studies are currently being conducted, in renal transplant recipients under immunosuppressive therapy and in allogeneic stem cell transplantation patients, to assess the sensitivity of the optimized CMV-specific IFN-γ ELISpot assay in this clinical context and to correlate results of the assay to clinical outcome.

## Conclusions

Altogether, this optimized, standardized and user-friendly CMV-specific IFN-γ ELISpot assay meet all the conditions of a sensitive and reliable diagnostic test for the monitoring of the functionality of CMV-specific CMI. Beside its validation in healthy individuals (this study), its suitability to assess CMV-specific cell-mediated immunity in a cohort of hemodialysis patients representative of patients prior to transplantation has been demonstrated [49]. Further validation of the assay in immunocompromised patients, such as transplant recipients is necessary to determine its suitability in assisting clinicians to evaluate the risk of CMV reactivation and disease, and possibly individualize the therapeutic management of patients.

## Additional files

Additional File 1: Assay variability. Intra-assay (**Table S1.**), inter-assay (**Table S2.**), inter-operator (**Table S3.**) and inter-site (**Table S4.**) variability was assessed as described in the respective legends. (PDF 128 kb)
Additional File 2: Positivity cut-off definition. Results obtained using the optimized IFN-γ ELISpot assay on PBMC isolated from whole blood of 45 healthy donors (Table 1 and Fig. 8) were used to generate precision profiles for each IE-1 and pp65 stimulation. Limits of quantitation (LoQ) for IE-1 and pp65 were 8.6 and 7.1 SFC/200,000 PBMC respectively. LoQ calculated on a cohort of 124 hemodialysis patients [49] were in a similar range (7.8 and 8.3 for IE-1 and pp65 respectively). Therefore, a positivity cut-off of 10 SFC/200,000 PBMC was chosen for the standardized assay. In addition, intra-assay standard deviation (SD) within both cohorts of healthy donors (n = 45) and hemodialysis patients (n = 124) for stimulated and unstimulated measurements was the basis for the calculation of a criterion that the ratio of geometric means of stimulated to unstimulated values is at least 2.5. Finally, considering a test result as positive when geometric mean for at least one of the IE-1 or pp65 stimulated approach is ≥ 10 SFC/200,000 PBMC, positive agreement (sensitivity) and negative agreement (specificity) of the optimized IFN-γ ELISpot test results with CMV serology within the collective of 45 healthy donors was 97% and 85% respectively (Fig. 8). (PDF 73 kb)
Additional File 3: Gating strategy of flow cytometry analyses following surface marker (CD3, CD4, CD8, CD56) and intracellular IFN-γ staining. PBMC (4 replicates each) from six CMV-seropositive healthy donors were stimulated with T-activated® pp65 and IE-1 antigens for 6 h, as indicated in the legend to Fig. 9. (**A**) Cells were first gated on single events in a Forward Scatter-Area (FSC-A) x FSC-Height (FSC-H) dot plot (Gating #1). Based on FSC-A x Side Scatter-Area (SSC-A) properties, a gate was next set around lymphocytes (Gating #2). By plotting CD3 against CD56, CD56 single positive cells (Gating #3), CD3/CD56 double positive cells (Gating #4) and CD3 single positive cells (Gating #5) were separated. CD3 single positive cells (Gating #5) were further subdivided into CD3/CD4 double positive (Gating #6) and CD3/CD8 double positive cells (Gating #7). (**B**) Representative dot plots (1 out of 4 replicates) of stained PBMC of donor d290. In each plot, percentage value in gated area is the mean frequency (from 4 replicates) of IFN-γ-expressing CD3^+^CD4^+^ (Th), CD3^+^CD8^+^ (CTL), CD3^−^CD56^+^ (NK) and CD3^+^CD56^+^ (NKT-like) cells. (PDF 351 kb)

## Abbreviations

CMI: Cell-mediated immunity
CMV: Cytomegalovirus
CTL: Cytotoxic T lymphocyte
CV: Coefficient of variation
ELISA: Enzyme-linked immunosorbent assay
ELISpot: Enzyme-linked immunospot
HLA: Human leukocyte antigen
ICS: Intracellular cytokine staining
IE-1: Immediate early-1 protein
IFN-γ: Interferon gamma
LoQ: Limit of quantitation
NK: Natural killer cell
NKT: Natural killer T cell
PBMC: Peripheral blood mononuclear cell
pp65: 65 kDa lower matrix phosphoprotein
RSD: Relative standard deviation
SD: Standard deviation
SFC: Spot-forming cell
Th: T helper cell
