# Supplementary material for: An optimized IFN-γ ELISpot assay for the sensitive and standardized monitoring of CMV protein-reactive effector cells of cell-mediated immunity
Source: BMC Immunol. 2017 Mar 7;18:14. doi: 10.1186/s12865-017-0195-y (PMC5339961; doi:10.1186/s12865-017-0195-y)
Supplement: Additional File 1: — Assay variability. Intra-assay (Table S1.), inter-assay (Table S2.), inter-operator (Table S3.) and inter-site (Table S4.) variability was assessed as described in the respective legends. (PDF 128 kb) [file 12865_2017_195_MOESM1_ESM.pdf]

Table 1. Intra-assay variability

Neg.	Measurement 1	Measurement 2	SD	CV in %
d044	0.00	0.00	0.00	n.d.
d120	2.25	2.00	0.18	n.d.
d172	10.50	0.75	6.89	n.d.
**IE-1**	**Measurement 1**	**Measurement 2**	**SD**	**CV in %**
d044	3.00	9.00	4.24	n.d.
d120	63.50	60.00	2.47	4.01
d172	108.00	88.25	13.97	14.23
**pp65**	**Measurement 1**	**Measurement 2**	**SD**	**CV in %**
d044	192.75	182.25	7.42	3.96
d120	244.00	239.00	3.54	1.46
d172	975.25	1061.50	60.99	5.99

Two ELISpot measurements were performed by the same operator, on unstimulated PBMC (neg.) and on PBMC stimulated with T-activated® IE-1 and pp65, using PBMC from three CMV-seropositive healthy donors (d044, d120, d172). Mean SFC / 200,000 PBMC and standard deviation (SD) from quadruplicates are shown. Coefficient of variation (CV) for values < 10 SFC / 200,000 PBMC were not calculated (see text). CV intra-assay was below 15%.

Table 2. Inter-assay variability

Neg.	Measurement 1	Measurement 2	Measurement 3	SD	CV in %
d120	0.25	0.00	0.25	0.14	n.d.
d172	0.25	0.00	0.00	0.14	n.d.
d241	0.00	0.00	1.00	0.58	n.d.
**IE-1**	**Measurement 1**	**Measurement 2**	**Measurement 3**	**SD**	**CV in %**
d120	12.25	16.00	12.25	2.17	16.04
d172	40.25	31.50	44.75	6.74	17.35
d241	4.75	12.50	6.25	4.11	n.d.
**pp65**	**Measurement 1**	**Measurement 2**	**Measurement 3**	**SD**	**CV in %**
d120	124.25	114.75	88.75	18.38	16.82
d172	703.00	580.50	757.25	90.54	13.31
d241	49.50	60.50	38.75	10.88	21.93

Three independent ELISpot measurements were performed by the same operator, at one location and using the same reagent batches and equipment, on PBMC from the same three CMV-seropositive healthy donors (d120, d172, d241). In each assay, PBMC were left unstimulated (neg.) or were stimulated with T-activated® IE-1 and pp65. Mean SFC / 200,000 PBMC and standard deviation (SD) from quadruplicates are shown. Coefficient of variation (CV) for values < 10 SFC / 200,000 PBMC were not calculated (see text). CV inter-assay did not exceed 22%.

Table 3. Inter-operator variability

Neg.	Operator 1	Operator 2	Operator 3	SD	CV in %
d172	0.50	0.25	0.50	0.14	n.d.
d204	0.13	0.00	0.00	0.07	n.d.
d254	0.25	0.25	0.50	0.14	n.d.
**IE-1**	**Operator 1**	**Operator 2**	**Operator 3**	**SD**	**CV in %**
d172	45.75	45.38	36.13	5.45	12.85
d204	4.50	7.50	4.25	1.81	n.d.
d254	750.75	901.75	853.63	77.14	9.23
**pp65**	**Operator 1**	**Operator 2**	**Operator 3**	**SD**	**CV in %**
d172	1059.13	1093.00	1044.13	25.04	2.35
d204	230.38	278.38	195.38	41.67	17.75
d254	603.63	596.88	668.00	39.26	6.30

PBMC from three CMV-seropositive healthy donors (d172, d204, d254) were isolated and assayed in parallel (optimized protocol) by three different operators at one location, using the same reagent batches and equipment. Each assay was performed on unstimulated PBMC (neg.) and on PBMC stimulated with T-activated® IE-1 and pp65. Mean SFC / 200,000 PBMC and standard deviation (SD) from quadruplicates are shown. Coefficient of variation (CV) for values < 10 SFC / 200,000 PBMC were not calculated (see text). CV inter-operator did not exceed 18%.

Table 4. Inter-site variability

IE-1	# 1	# 2	# 3	# 4	# 5	# 6	# 7	SD	CV in %
d1	21.25	11.00	37.50	27.50	28.75	30.75	17.75	8.21	32.95
d2	14.50	16.25	28.50	19.50	15.00	15.50	12.75	4.90	28.14
d3	16.50	12.50	29.00	12.25	22.50	29.60	11.00	7.39	38.81
**pp65**	**# 1**	**# 2**	**# 3**	**# 4**	**# 5**	**# 6**	**# 7**	**SD**	**CV in %**
d1	209.25	153.00	333.75	274.00	212.00	286.75	151.75	64.04	27.66
d2	478.75	448.50	848.25	650.25	567.75	636.75	373.25	146.43	25.60
d3	689.00	529.25	790.25	784.50	683.50	791.00	458.75	123.64	18.31

ELISpot assays were performed at 4 different locations in Germany by 7 different operators (measurements #1-7), following stimulation with T-activated® pp65 and IE-1 of PBMC isolated from the same blood donation of three CMV-seropositive healthy individuals (d1-d3). Mean SFC / 200,000 PBMC and standard deviation (SD) from quadruplicates are shown. CV inter-site for IE-1 and pp65 stimulations was below 39% and 28% respectively.
