# Supplementary figures and images for: An optimized IFN-γ ELISpot assay for the sensitive and standardized monitoring of CMV protein-reactive effector cells of cell-mediated immunity
Source: BMC Immunol. 2017 Mar 7;18:14. doi: 10.1186/s12865-017-0195-y (PMC5339961; doi:10.1186/s12865-017-0195-y)

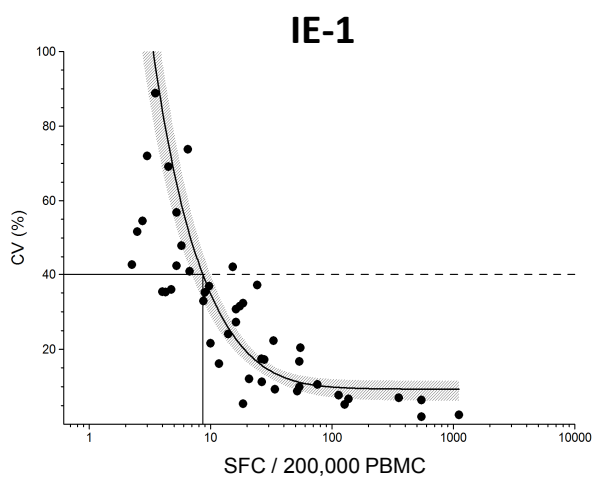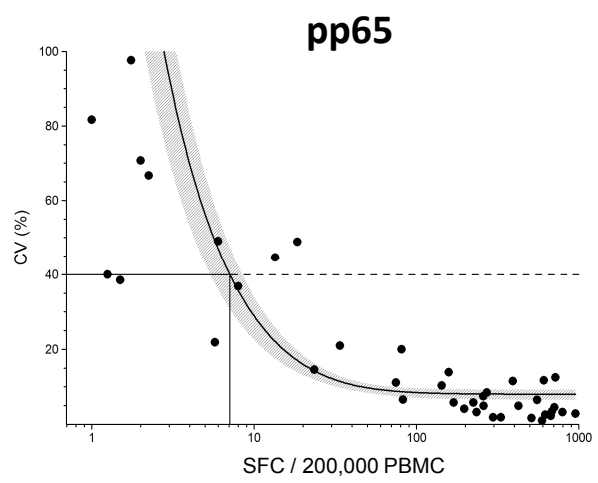

Additional File 2 (Barabas et al.)

Supplement: Additional File 2: — Positivity cut-off definition. Results obtained using the optimized IFN-γ ELISpot assay on PBMC isolated from whole blood of 45 healthy donors (Table 1 and Fig. 8) were used to generate precision profiles for each IE-1 and pp65 stimulation. Limits of quantitation (LoQ) for IE-1 and pp65 were 8.6 and 7.1 SFC/200,000 PBMC respectively. LoQ calculated on a cohort of 124 hemodialysis patients [49] were in a similar range (7.8 and 8.3 for IE-1 and pp65 respectively). Therefore, a positivity cut-off of 10 SFC/200,000 PBMC was chosen for the standardized assay. In addition, intra-assay standard deviation (SD) within both cohorts of healthy donors (n = 45) and hemodialysis patients (n = 124) for stimulated and unstimulated measurements was the basis for the calculation of a criterion that the ratio of geometric means of stimulated to unstimulated values is at least 2.5. Finally, considering a test result as positive when geometric mean for at least one of the IE-1 or pp65 stimulated approach is ≥ 10 SFC/200,000 PBMC, positive agreement (sensitivity) and negative agreement (specificity) of the optimized IFN-γ ELISpot test results with CMV serology within the collective of 45 healthy donors was 97% and 85% respectively (Fig. 8). (PDF 73 kb) [file 12865_2017_195_MOESM2_ESM.pdf]

**A****d290**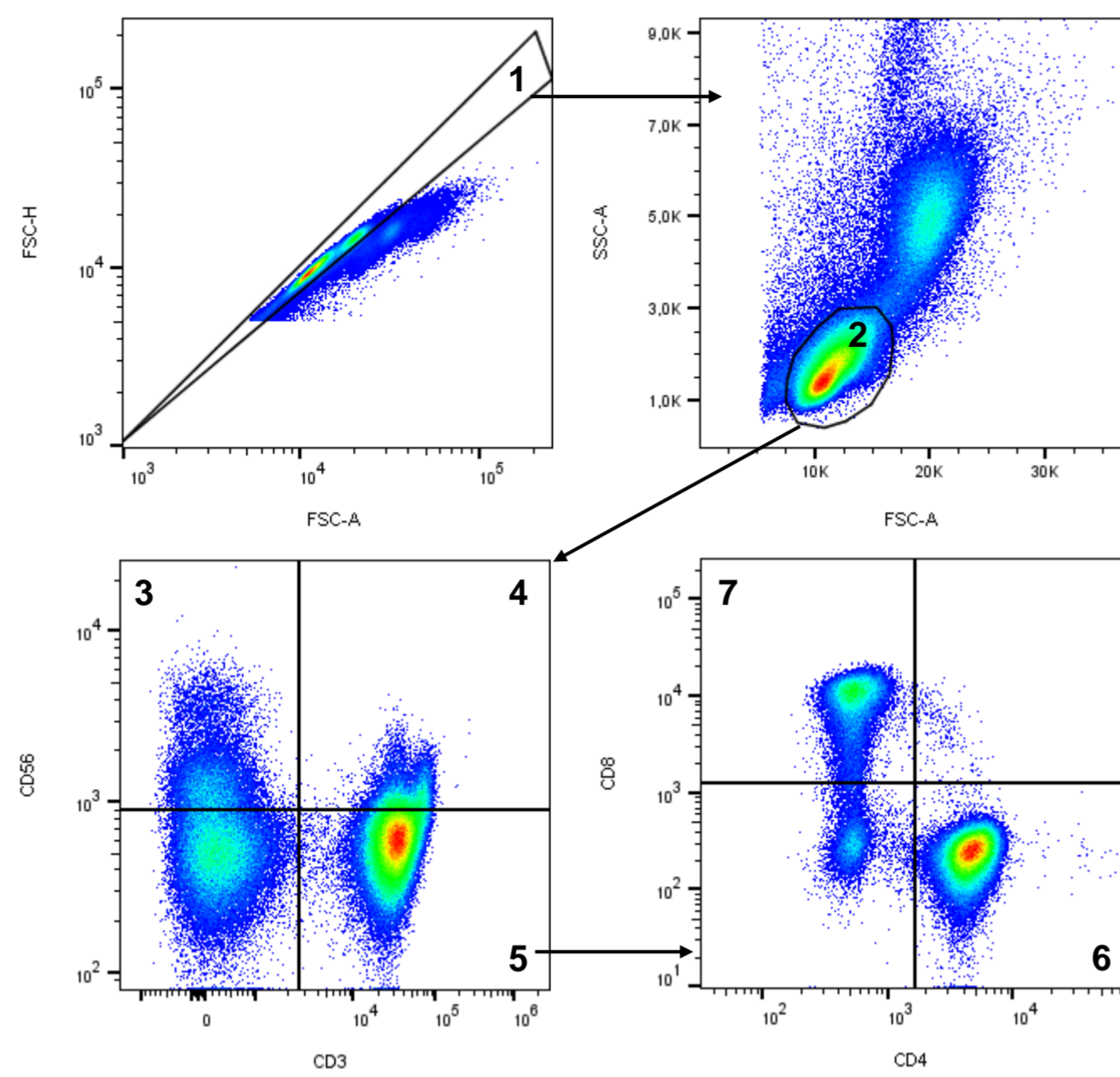**B****d290**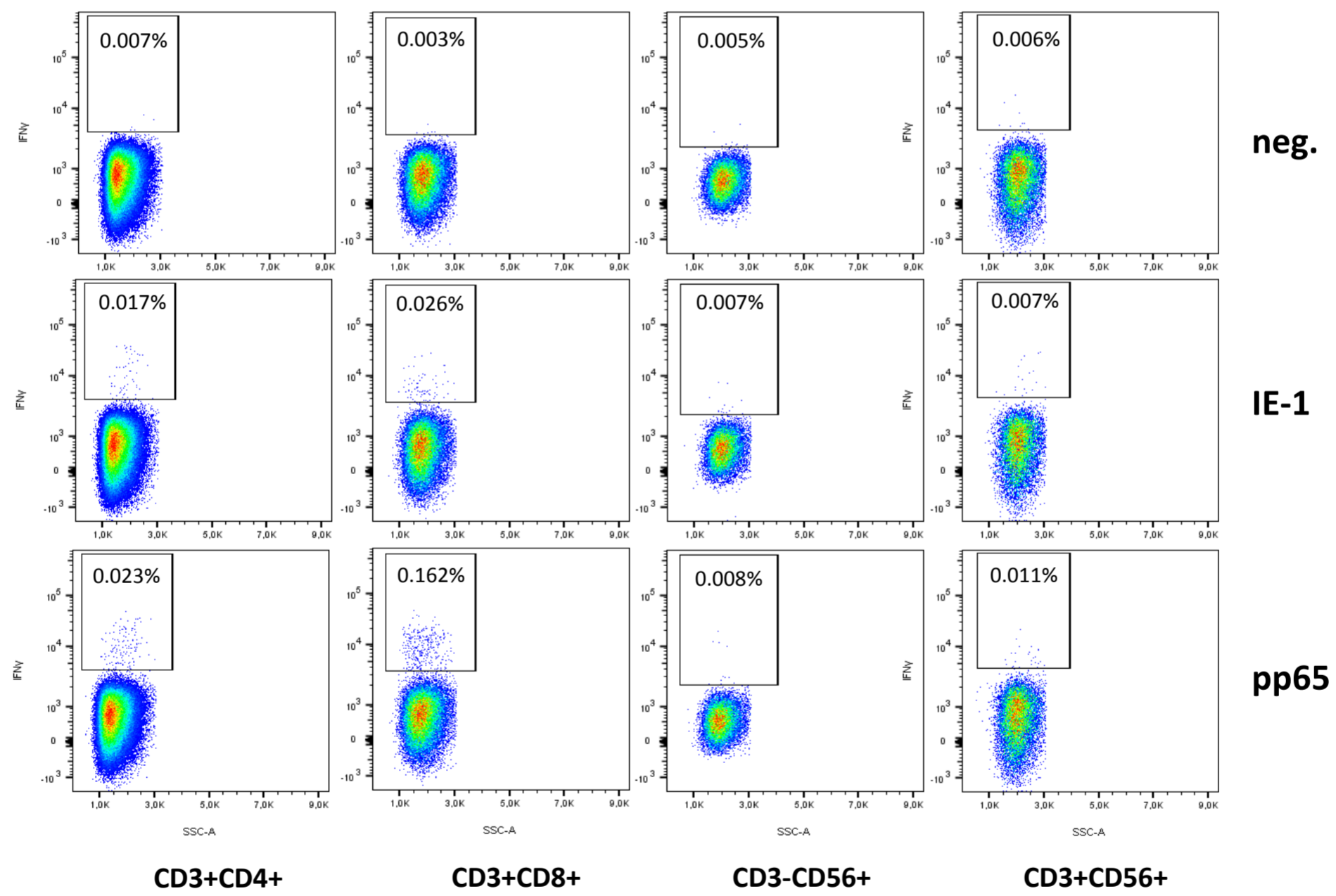

Supplement: Additional File 3: — Gating strategy of flow cytometry analyses following surface marker (CD3, CD4, CD8, CD56) and intracellular IFN-γ staining. PBMC (4 replicates each) from six CMV-seropositive healthy donors were stimulated with T-activated® pp65 and IE-1 antigens for 6 h, as indicated in the legend to Fig. 9. (A) Cells were first gated on single events in a Forward Scatter-Area (FSC-A) x FSC-Height (FSC-H) dot plot (Gating #1). Based on FSC-A x Side Scatter-Area (SSC-A) properties, a gate was next set around lymphocytes (Gating #2). By plotting CD3 against CD56, CD56 single positive cells (Gating #3), CD3/CD56 double positive cells (Gating #4) and CD3 single positive cells (Gating #5) were separated. CD3 single positive cells (Gating #5) were further subdivided into CD3/CD4 double positive (Gating #6) and CD3/CD8 double positive cells (Gating #7). (B) Representative dot plots (1 out of 4 replicates) of stained PBMC of donor d290. In each plot, percentage value in gated area is the mean frequency (from 4 replicates) of IFN-γ-expressing CD3+CD4+ (Th), CD3+CD8+ (CTL), CD3−CD56+ (NK) and CD3+CD56+ (NKT-like) cells. (PDF 351 kb) [file 12865_2017_195_MOESM3_ESM.pdf]
